# Exercise‐induced anaphylaxis triggered by bird‐egg syndrome: unraveling the link between egg yolk and bird allergens

**DOI:** 10.1111/ddg.15852

**Published:** 2025-08-25

**Authors:** Andrea Nolting, Peter Schmid‐Grendelmeier, Carole Guillet

**Affiliations:** ^1^ Allergy Unit Department of Dermatology University Hospital Zurich Zurich Switzerland; ^2^ Faculty of Medicine University of Zurich Zurich Switzerland; ^3^ Christine Kühne Center for Allergy Research and Education (CK‐CARE) Davos Switzerland

Dear Editors,

An 18‐year‐old female patient was referred for evaluation following a suspected exercise‐induced anaphylactic reaction. The episode occurred one hour after she had consumed a quiche containing sheep cheese and subsequently went for a run. After about 10 minutes, she developed a dry cough and sweating, followed by shortness of breath and generalized urticaria. No emergency medical attention was sought, as the breathing difficulties subsided quickly, and other symptoms resolved within 8 hours. The episode was perceived as highly threatening by the patient. For the past few years, she had experienced gastrointestinal symptoms (cramps, diarrhea) and oral itching after meals but could not identify the triggers. Keeping a food diary following the above‐mentioned episode led her to suspect egg as the culprit, as she experienced symptoms after consuming both raw and cooked egg, including egg white and yolk. The patient had a known allergy to cat hair but denied symptoms of pollinosis. The patient had been exposed to pet budgerigars and rabbits since childhood and reported sneezing and itchy eyes when near the birdcage. She had no other pre‐existing conditions.

Allergy testing was performed, including prick tests for various pollen, dust mites, molds, egg (both white and yolk), and avian feathers. Results were positive for egg white, egg yolk, total egg, house dust mites, and some pollen. Skin prick test results for egg, egg white, and egg yolk are shown in Figure 1. Serological testing revealed elevated total IgE (172 kU/l) and specific IgE against egg white and egg yolk (α‐livetin, Gal d5). Sensitization patterns are shown in Table 1.

**FIGURE 1:**
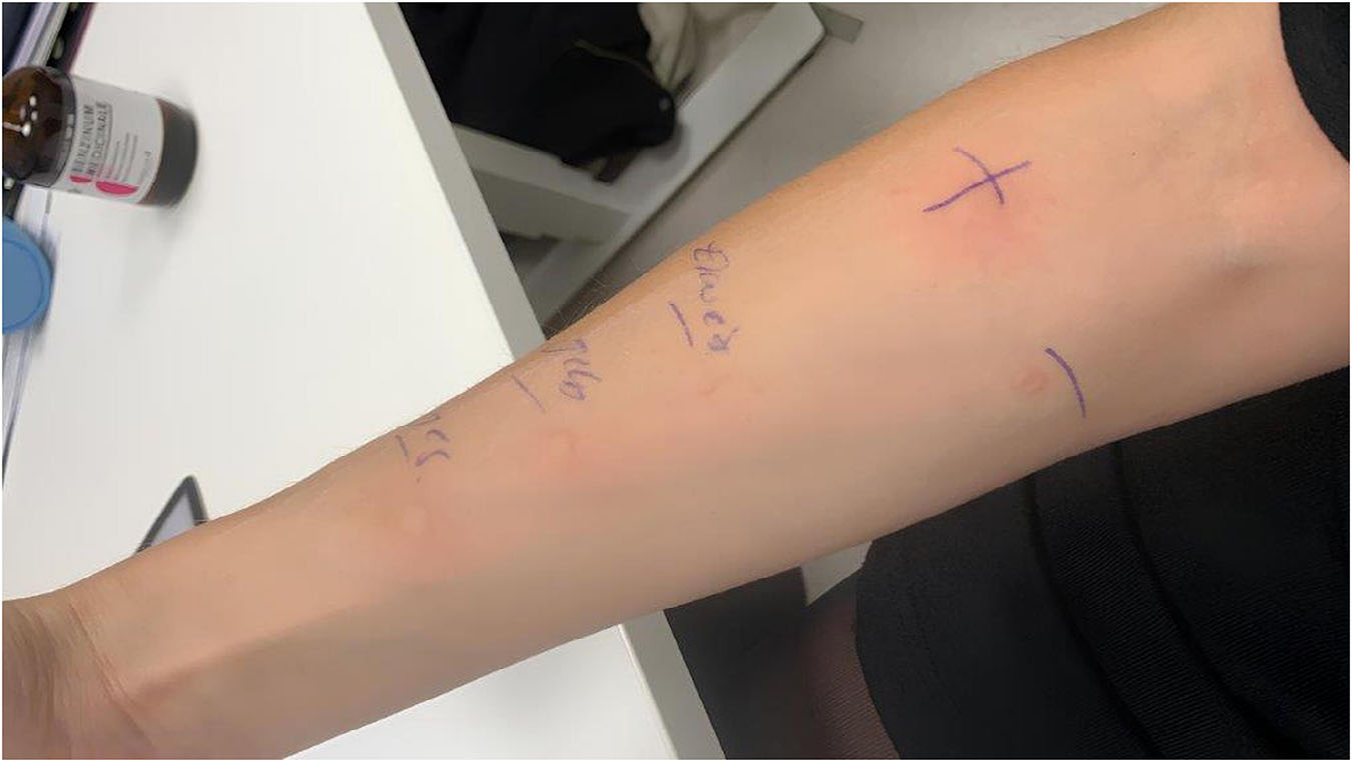
Prick testing for various components of chicken egg. Positive reactions are seen with standard prick test solutions, from top to bottom: egg white, egg yolk, and whole egg. Histamine (marked with a “+”) serves as the positive control.

**TABLE 1:** Serological work‐up in an 18‐year‐old female patient with chicken egg allergy.

Parameter	Result
Total‐IgE	172 kU/l
Whole egg extract	3.9 kU/l (NL: < 0.1 kU/l)
Ovomucoid (Gal d1)	< 0.1 kU/l
Ovalbumin (Gal d2)	< 0.1 kU/l
Egg yolk (Gal d5)	0.56 kU/l
House dust mites	
*Dermatophagoides pteronyssinus*	8.29 kU/l
*Dermatophagoides farinae*	18.5 kU/l
Pollen	
Timothy grass pollen	1.45 kU/l
Birch pollen	2.56 kU/l

However, specific IgE for ovomucoid (Gal d1) and ovalbumin (Gal d2) were not elevated. Testing for poultry, milk or wheat allergies was not performed since the patient reported oral tolerance in daily life.

Based on the typical symptoms following egg consumption, positive prick tests, and detection of specific IgE antibodies, we diagnosed egg allergy with possible exercise‐induced anaphylaxis. Positive IgE for Gal d5 and rhinoconjunctival symptoms when exposed to budgerigars indicated potential bird‐egg syndrome even though skin prick tests with avian feathers were negative and poultry was consumed without problems. The patient was advised to avoid all foods containing egg white or yolk and educated on early symptom recognition and quick response to potential triggers. She was specifically instructed to avoid both raw and cooked egg, including traces of egg in processed foods. She was provided with an emergency kit containing an antihistamine and corticosteroid in case of accidental ingestion. It was also recommended that an epinephrine auto‐injector be carried at all times. Oral immunotherapy for egg allergy was not advised as it is still mainly reserved for children.^1^, ^2^

Egg allergy in adults is rare, with a prevalence of about 0.1%. In children, however, it is the second most common food allergy, often resolving by adolescence.^3^, ^4^ Symptoms vary but often involve gastrointestinal reactions, with skin reactions or severe anaphylactic responses also possible.^5^ Egg white contains four major allergens: ovomucoid (Gal d1), ovalbumin (Gal d2), ovotransferrin (Gal d3), and lysozyme (Gal d4). For egg yolk, chicken serum albumin (Gal d5) and YGP42 (Gal d6) are the primary allergens.^6^ Our patient had elevated IgE for total egg extract and specific IgE for Gal d5, indicating an allergy to egg yolk. Although common egg white allergens were not elevated, the patient's intolerance of egg white suggests a potential sensitization to additional allergens.

Studies conducted in the past few years link egg yolk allergies, characterized by elevated IgE against chicken serum albumin (Gald d5), to poultry meat and bird dust allergies, in other words the bird‐egg syndrome.^2^ Our patient was tolerant of poultry as food, but experienced rhinoconjunctivitis in proximity to their bird cage, which could potentially be considered as a mechanism of sensitization to egg yolk.

Anaphylaxis can be exacerbated by various triggers, including exercise, stress, infections, medications, and alcohol.^7^ Although an exercise provocation test is considered the gold standard for confirming food‐dependent exercise‐induced anaphylaxis, we refrained from performing it due to the clear correlation between egg consumption and symptoms, as well as the potential burden and risk to the patient. Additionally, the patient experienced symptoms, though milder, without exercise, further supporting the primary diagnosis of egg allergy. Diagnosis of egg allergy necessitates significant dietary changes, which can limit food variety, reduce quality of life, and increase anxiety about allergic reactions.^8^ As the prevalence of food allergies ‐ including egg allergies ‐ in adulthood continues to rise, public awareness, clear labeling, and prevention play a key role in avoiding anaphylactic incidents, improving the quality of life for allergy sufferers, and lowering healthcare costs.

## CONFLICT OF INTEREST STATEMENT

P.S.‐G. has received honoraria for lectures and advisory boards from Euroimmun and ThermoFisher Diagnostics. A.N. and C.G. have declared that they have no potential conflicts of interest.
